# The ‘why’ of radiology and related issues

**DOI:** 10.4103/0971-3026.41824

**Published:** 2008-08

**Authors:** Bhavin Jankharia

**Affiliations:** Indian Journal of Radiology and Imaging, Bhaveshwar Vihar, 383 Sardar V. P. Rd, Prarthana Samaj, Mumbai - 400 004, India. E-mail: editor@ijri.org

In the practice of radiology, there really is just one question that needs to be answered each time the radiologist performs or reports a study. The question is ‘Why?’–‘Why am I performing this study?’ or ‘Why was this study requested?’

If the answer to this question is clear, almost everything else falls into place. One doesn't have to be the world's greatest radiologist to do good work; when doing a study or interpreting imaging findings, what is needed is common sense and the ability to understand what the question is that the referring doctor wants answered.

On any given day, 80% of all studies performed or interpreted are routine, for example, reporting on chest radiographs, routine obstetric USG scans, CT of brain in stroke and headaches, etc., demanding no significant additional thought from the radiologist. However, in about 20% of the cases the radiologist will need to stop and ask: Why was this study asked for/done? What did the referring doctor want? What exactly is the question that needs to be answered? Often, to answer these questions, it is necessary to call the referring doctor to understand the relevant issues, especially if a detailed history is not available. Sometimes it is necessary to go through all the old papers and investigation reports and, if necessary, to talk to the patient or even re-image if the correct scanning protocol has not been followed in an earlier study. If at the end of all this, one still doesn't know why the study was done or if, because of the absence of relevant history, one is unable to interpret the study, then the right thing to do is to describe the findings in the report and honestly confess that the interpretation of these findings is difficult in the absence of additional relevant information.

Looking around, it is clear that the professionally successful radiologists are the ones who, having figured this out, are able to satisfy the clinical needs of their referring doctors. These are the radiologists to whom all the complicated cases are referred and to whom the referring doctors go when they themselves have problems.

Residents, senior registrars, lecturers, and those who immediately plunge into practice after their MD or DNB rarely understand this! I have seen brilliant young radiology residents who know everything there is to know about “Radiological signs of…” or “Imaging findings of…” but who pay scant attention to the ‘why’! In fact, in today's day and age of easy access to information, it is not necessary to know everything about a disease or its radiological signs or to know all the syndromes and measurements. What is truly important in today's connected world is to know where to look for the answers: whether in carefully archived articles, in textbooks, or in Pubmed, Google Scholar, or Wikipedia. Since the need to remember everything has actually reduced due to this easy access to information, we now need to use our grey cells more efficiently by being more involved in the clinical situations and issues related to the patients who come to us.

This applies to speakers and teachers as well. It is all very well to give a lecture on multiple sclerosis (MS) and show 50 slides of the various MRI appearances of MS. This is no big deal! All this information is easily available in textbooks and in umpteen review articles, all freely available at the AJR or Radiographics sites. What the speakers and teachers need to bring to the table are the clinical issues related to MS: what are the common problems that patients come with and what kind of information is expected from the radiologist, given a particular clinical situation and presentation. The students and residents should also expect and demand more from their teachers and from speakers than mere regurgitation of material from textbooks.

Things were different in the days of x-rays, simple radiographic procedures, and basic USG, when the questions that needed to be answered were simpler and often easily understandable. With plain radiographs, even if the radiologists screwed up, very often the physicians, orthopedic surgeons, etc. were able to interpret the radiographs and manage things. Today, when even radiologists have trouble keeping their skills up-to-date, to expect the referring doctor to be able to correctly interpret complex imaging findings is unrealistic, and the radiologists' responsibilities have increased that much more.

That we need to be more involved clinically is something that Mr. Ragavan also talks about in ‘Radiology in India: The Next Decade.’ This is a new series that we have started, edited by Dr Sanjeev Mani, where over the next 2–3 issues, we will have eminent radiologists and industry experts airing their thoughts on this subject. 

Paradoxically, we have also seen the rise of teleradiology, both in India as well as in the rest of the world, something that Dr Arjun Kalyanpur is very upbeat about in his article in the same series. However, the ‘why’ of radiology and teleradiology are at opposite ends of the radiology spectrum. Teleradiology takes us back to the early days of radiology, with radiologists sitting in dark dungeon-like offices, reporting radiographs without any concern for the clinical setting. Interpreting the imaging findings of patients who are physically and figuratively very far away, with no clinical interaction, may be a good way to provide a commodity service and to earn money, but it is not a good way to practice radiology. There are of course exceptions, for example, when teleradiology is used for obtaining expert, second opinions or for nighttime, emergency coverage, where the questions to be answered are much simpler. But, overall, our growth as radiologists will come from our ability to understand the clinical issues surrounding our patients and not by interpreting ‘unknown’ images.

This also brings us to the issue of subspecialization. To be useful to our clinical colleagues, we must be able to speak their language. To be able to do this, we must subspecialize in specific areas, whether it be neuroradiology, head and neck, cardiac, musculoskeletal, etc. It is not necessary to be a single-organ subspecialist in our country as yet, since the economic and social realities make this difficult except in some academic institutions. Yet to expect to be able to handle all organ systems is also stupid. Currently, the best solution would be to focus on 2–3 areas, eg, neuroradiology and head and neck; chest and cardiac imaging; abdominal imaging along with pelvic imaging; or women's imaging, which would include gynecologic, obstetric, and breast imaging. 

The advent of subspecialization in radiology also means that radiologists who have subspecialized in different organ systems will have to work together in groups. In academic institutes, this is easily done. Sadly, however, except in 2–3 institutes, radiologists in our country still want to do everything or rotate through every modality and organ system. In private hospitals and private practice, the only way to subspecialize is to work in cohesive groups. This allows the groups to gain tremendous depth of knowledge, respect, and a good patient load; in addition, the radiologists' quality of life will also improve significantly: there is no need to be on call 24 × 7; one can take holidays or take time off when required. Even if it means earning a shade less, over the long term, group practice with subspecialization works out to be a much better deal than working solo and trying to do everything. This is how I currently practice radiology and this, in my view, is the future of radiology and radiologists in our country. 

Let me summarize. All our radiology reports need to answer the ‘why’ question. To do this, we must be thorough with our understanding of the clinical situation and the answers being sought by our clinical colleagues. For this to happen well, we must subspecialize and be able to speak the same language as our clinical colleagues. Subspecialization inherently means that we need to work with other radiology subspecialists in a group practice; both academically and in general, this would automatically lead to a better quality of life.

